# Predicting Environmental and Ecological Drivers of Human Population Structure

**DOI:** 10.1093/molbev/msad094

**Published:** 2023-05-05

**Authors:** Evlyn Pless, Anders M Eckburg, Brenna M Henn

**Affiliations:** Department of Anthropology, Center for Population Biology, University of California, Davis, CA; Department of Anthropology, Center for Population Biology, University of California, Davis, CA; Department of Anthropology, Center for Population Biology, University of California, Davis, CA; UC Davis Genome Center, University of California, Davis, CA

**Keywords:** migration, MAPS, SPRUCE, random forest, gene flow, barriers

## Abstract

Landscape, climate, and culture can all structure human populations, but few existing methods are designed to simultaneously disentangle among a large number of variables in explaining genetic patterns. We developed a machine learning method for identifying the variables which best explain migration rates, as measured by the coalescent-based program MAPS that uses shared identical by descent tracts to infer spatial migration across a region of interest. We applied our method to 30 human populations in eastern Africa with high-density single nucleotide polymorphism array data. The remarkable diversity of ethnicities, languages, and environments in this region offers a unique opportunity to explore the variables that shape migration and genetic structure. We explored more than 20 spatial variables relating to landscape, climate, and presence of tsetse flies. The full model explained ∼40% of the variance in migration rate over the past 56 generations. Precipitation, minimum temperature of the coldest month, and elevation were the variables with the highest impact. Among the three groups of tsetse flies, the most impactful was *fusca* which transmits livestock trypanosomiasis. We also tested for adaptation to high elevation among Ethiopian populations. We did not identify well-known genes related to high elevation, but we did find signatures of positive selection related to metabolism and disease. We conclude that the environment has influenced the migration and adaptation of human populations in eastern Africa; the remaining variance in structure is likely due in part to cultural or other factors not captured in our model.

## Introduction

There is great interest in the mechanisms that determine gene flow and cause population structure, but there are computational challenges to disentangling the many factors at play. In humans, there are a variety of complex and often correlated variables—land cover, climate, culture, and language—that all affect migration patterns and resulting population structure. In general, we know populations that are farther apart tend to exchange fewer migrants (“isolation by distance”; Wright 1943); however, gene flow does not always scale proportionally with geographic distance (Wang and Bradburd 2014). A mountainous region might limit gene flow between two human populations more than equally spaced populations at the same elevation, and the same idea applies to cultural barriers such as language. There are a variety of landscape genetics tools designed to find associations between spatial variables and gene flow (Manel et al. 2003). In landscape genetics, it is often useful to depict migration routes and barriers through “resistance surfaces,” in which each pixel has a value representing the difficulty of migrating across this pixel. Least cost path analysis or circuit theory (McRae et al. 2008) can then be used to calculate “resistance distances” between pairs of populations or individuals through these surfaces; these effective distances can then be associated with proxies for genetic distances to find correlations. Genetic connectivity can also be modeled directly from environmental data (Bouyer et al. 2015) and using a variety of statistical methods including linear mixed models (Row et al. 2017) and gravity modeling (Parsley et al. 2020). Another important advance is inferring migration surfaces (the inverse of resistance surfaces) from genetic distance data (EEMS) (Petkova et al. 2016) or from identical by descent (IBD) tracts shared between individuals (MAPS) (Al-Asadi et al. 2019); however, these models do not explicitly include environmental or other spatial variables.

Previously, Pless et al. (2021) developed an approach to infer landscape connectivity by integrating genetic and environmental data. This approach offered some important advantages not found in most other approaches, in particular, the ability to deal with 1) correlated predictor variables (environmental) and 2) flexible, nonlinear relationships between the predictor variables and the response variable (genetic distance) at different regions. A disadvantage of this model was that it used pairwise summary statistics such as F_ST_ (Rousset 1997) and Cavalli-Sforza & Edward's chord distance (Cavalli-Sforza and Edwards 1967) as proxies for genetic distance; these statistics make some limiting assumptions and can be biased by demography (Kalinowski 2002; Tumas et al. 2018). Additionally, it required the model to be based on pairwise distances among assumed populations or individuals, rather than using a single data entry for each unit of study (i.e., each population or individual). Although these features were necessary given the use case (clustered sampling of *Aedes aegypti* mosquitoes genotyped with 12 microsatellites), they made the model more difficult to use and interpret. The method was also computationally intensive and relied on some specialized Geographic Information System (GIS) software packages that are not accessible to all population geneticists. In this manuscript, we adapt and improve this previous method to circumvent these limitations for high-resolution genomic data. Instead of using summary statistics, we infer a migration surface based on regions of individuals’ genomes that are inherited from a common ancestor. We then find what spatial variables predict migration using the machine learning tool “random forests” (Breiman 2001), in a new approach we call SPRUCE (Spatial Prediction using Random forest to Uncover Connectivity among Environments).

We applied SPRUCE to eastern Africa, a compelling case study for understanding human population structure and migration. Specifically, we focused on southwestern Ethiopia, Kenya, and northern Tanzania—which have the densest collection of publicly available genome-wide data. This region is rich with ecological diversity ranging from low-elevation deserts to forested mountains. Additionally, the history of human migration into the region is complex (Prendergast et al. 2019), contributing to the high diversity of ethnicities, languages, religions, and subsistence strategies found there today (López et al. 2021; Atkinson et al. 2022). Cattle herding was introduced from the Near East into this region by ∼4,000 years ago, and agriculturalists from western Africa, who spoke languages in the Bantu family, later expanded into the Kenya/Tanzania border region by ∼2,000 years ago (Hildebrand and Grillo 2012; Skoglund et al. 2017). Local hunter-gatherer populations have exhibited varied responses to these migrations, including reducing their range, moving, adopting farming with varying levels of gene flow, or coexisting with the pastoralists and agriculturalists (Gopalan et al. 2022). In the past as well as today, ethnic self-identification can lead to population structure through endogamy, that is nonrandom mating between groups.

Using SPRUCE, we investigated the role that environmental and ethnolinguistic variables play in shaping human migration in East Africa across three time intervals ranging from approximately 300–2,000 years ago. After finding environmental variables that likely impacted human migration in eastern Africa, we predicted that populations have adapted to microclimates in the region, which we explored further by running genome-wide selection scans on five populations from southwestern Ethiopia. Although these populations live within 70 km of one another, they practice a range of subsistence strategies and likely face different pressures in terms of pathogen exposure and high elevation, which can cause cardiovascular disease and pregnancy complications (Alkorta-Aranburu et al. 2012).

## New Approaches

### Developing SPRUCE for Use With High-Resolution Genomic Data

We improve our previously developed method for inferring landscape connectivity (Pless et al. 2021) for dense genomic data. A key difference between the previous method and the improved version of SPRUCE is how migration rates are calculated. In the original method, pairwise summary statistics (e.g., F_ST_) among all pairs of populations was used as a proxy for migration rates, which served as the response variable in a random forest regression. The predictor variables’ values were calculated by taking the mean of the pixel values found along straight lines connecting each pair of points in the first iteration, and in subsequent iterations “least-cost path” lines were used to represent more realistic migration paths through the landscape.

**Fig. 1.**
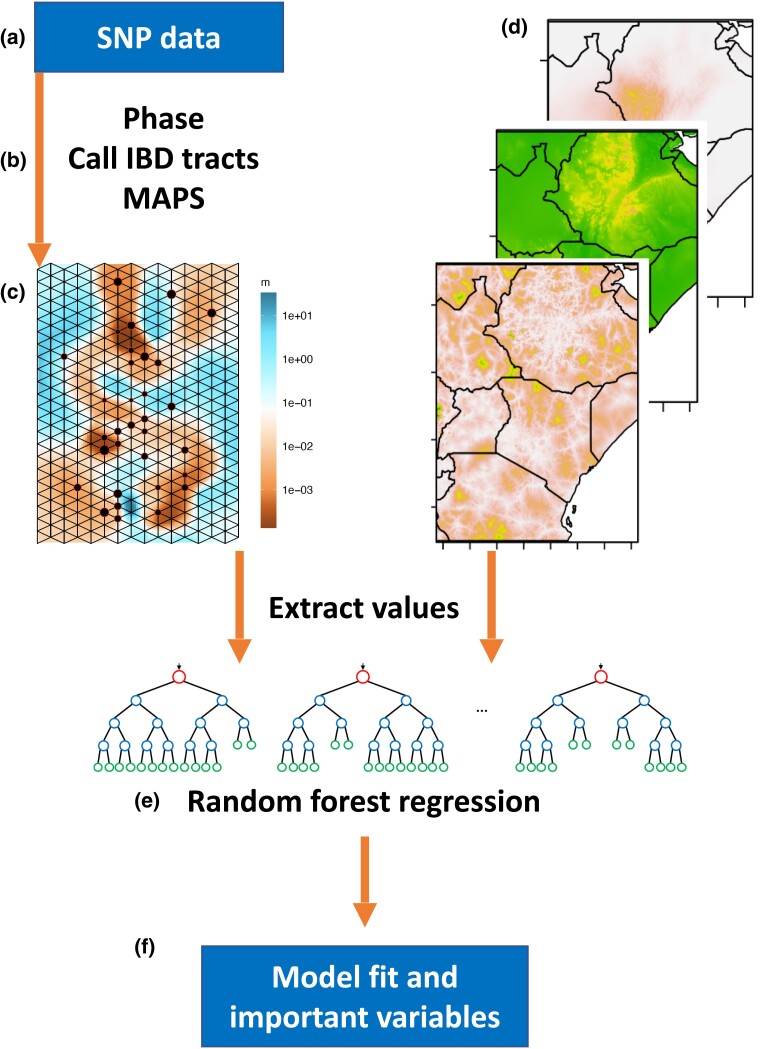
Overview of the SPRUCE pipeline for integrating genetic and spatial data to infer ecological drivers of human migration. The model uses a random forest framework to determine which spatial variables best predict migration rate which is inferred from sharing of IBD tracts among individuals using the program MAPS (Al-Asadi et al. 2019). More information, including explanation of each element of the pipeline (*a*–*f*), is provided in “New Approaches.”

In contrast, for SPRUCE, we calculate migration based on shared genomic patterns among individuals, rather than by using summary statistics such as F_ST_. We describe an overview of SPRUCE below and provide technical details in “Materials and Methods.” After processing and phasing the genomic data (fig. 1*a*), the first step in SPRUCE is to identify genomic regions which are shared IBD across each pair of individuals in the dataset (fig. 1*b*) (Al-Asadi et al. 2019). The number and length of shared IBD segments contain useful information for calculating relatedness and inferring migrations. IBD segments shorter than 2 centimorgans (cM) have a high false discovery rate (Browning and Browning 2011), so we examined IBD length categories of 2–4, 4–6, and >6 cM. Each of these categories contained >10,000 shared IBD tracts across the samples included in our analysis, and under a simplistic model of infinite population size, these length categories correspond to ∼56, 31, and 13 generations ago (see “Materials and Methods”). For each IBD length category, the user then calculates a matrix of shared genetic similarity among all individuals based on these tracts. The input to the MAPS software is this matrix and the geographic coordinates for each individual. The user also selects the grid size and number of Markov chain Monte Carlo (MCMC) iterations (see “Materials and Methods”). MAPS infers demographic parameters under a lattice and uses a Voronoi prior to regularizing parameters of the model (Al-Asadi et al. 2019). The inferred migration rate (migrants/generation) at each deme across the studied region provides the response variable in SPRUCE (fig. 1*c*). Migration rate “m” from MAPS is the average scaled rate into a given deme over the bidirectional migration occurring along all six edges in the lattice. The predictor variables are the values of the environment and other spatial variables corresponding to the location of each deme. Many variables of interest are available from free online datasets, such as CHELSA for worldwide climate data (Karger et al. 2017), and the deme-level values can be extracted with open-source tools such as gdal (fig. 1*d*) (GDAL/OGR Contributors 2021). We discuss our decision to use modern environmental datasets and the potential to incorporate historical climate reconstructions in the Discussion section.

We selected random forest to perform the regression between migration and spatial variables (fig. 1*e*). Random forest is well-suited because it can handle many inputs, including redundant or irrelevant variables, and it is less likely to overfit data since it builds each decision tree independently (Breiman 2001). It can handle multivariate regression and classification responses as well as mixed outcomes by growing a “multivariate forest” (Ishwaran et al. 2022), and it has an appropriate balance of flexibility (parameter tuning, weighting the bootstrapping) without requiring extensive machine learning expertise (Breiman 2001). There is also precedence for using it in ecology and landscape genetics (e.g., Brieuc et al. 2018). However, there is often a trade-off between model accuracy and interpretability, and the user may wish to substitute a less flexible but more interpretable model than a random forest, such as multivariate regression (James et al. 2021).

SPRUCE is suitable for genomic datasets that are dense enough to accurately identify IBD regions; > 400,000 single nucleotide polymorphisms (SNPs) is a reasonable, conservative threshold for humans (Browning and Browning 2011). We recommend applying the model to geographic regions where genetic samples are well-distributed, to capture variation in migration and environment. SPRUCE can accommodate a large number of diverse predictor variables, including categorical data (e.g., language) and correlated datasets (e.g., elevation and temperature). Overall, SPRUCE provides a straightforward approach for investigating which environmental variables contribute to migration rate, and how much variance these variables explain. We provide a test case including 10-fold cross-validation investigating how 25 spatial variables shape human migration in eastern Africa.

## Results

### SPRUCE Model

**Fig. 2.**
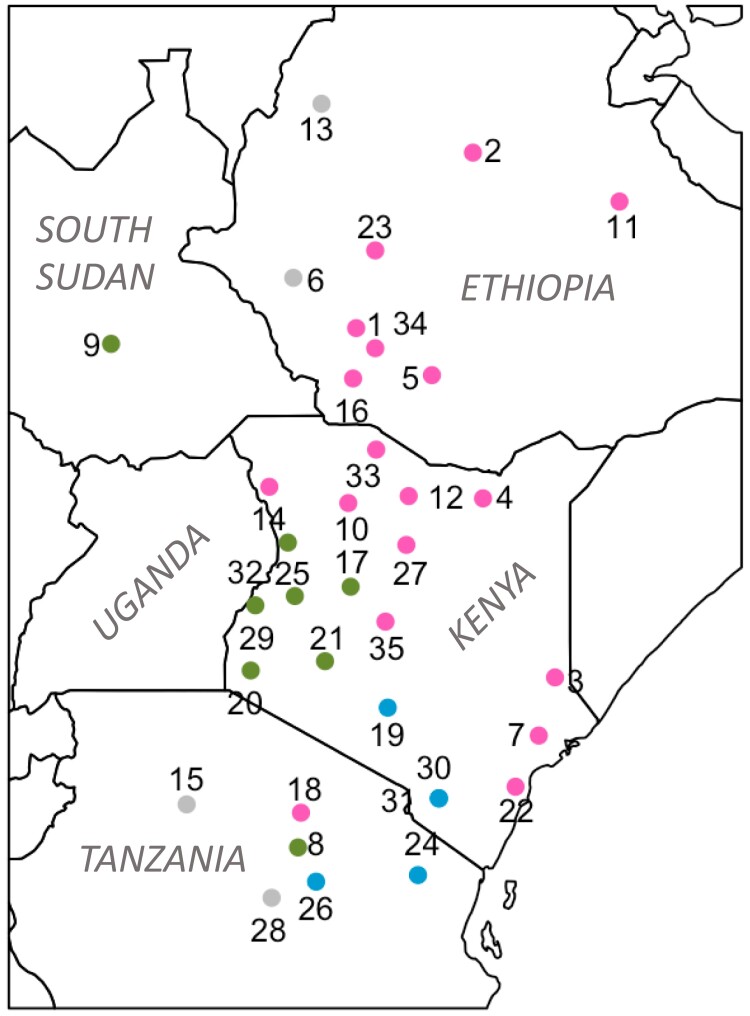
Geographic locations of genetic samples within eastern Africa included in the SPRUCE analysis (35 population locations, 492 individuals). Colors correspond to language family of the population (a variable included in the model as a proxy for language and culture): pink = Afro-Asiatic, blue = Niger-Congo, green = Nilo-Saharan, grey = isolate (6. Chabu, 13. Gumuz) or Khoisan (15. Hadza, 28. Sandawe). Numbers correspond to supplementary table S1, Supplementary Material online.

**Fig. 3.**
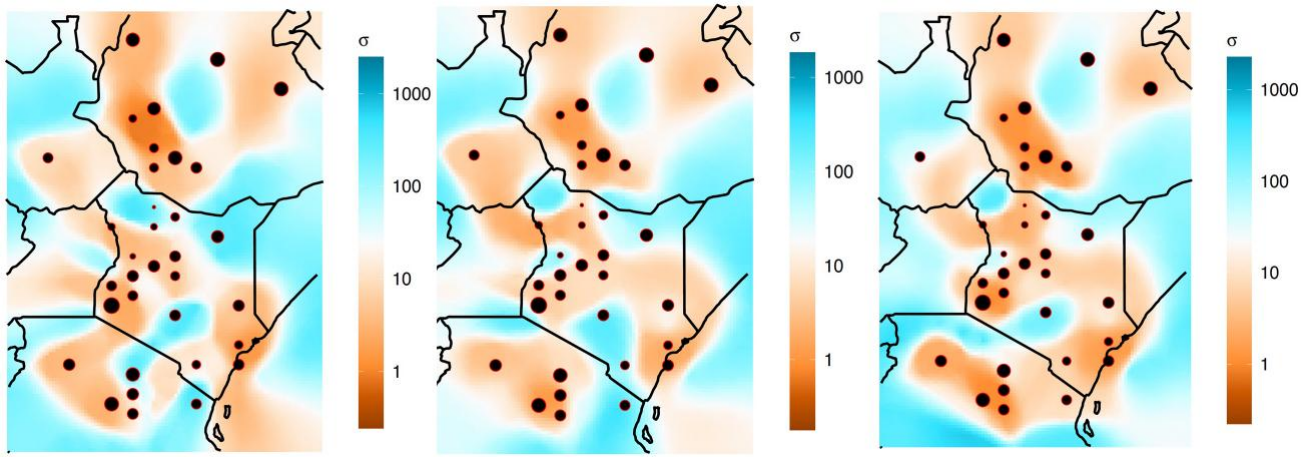
Inferred dispersal surfaces based on shared IBD tracts using the program MAPS (Al-Asadi et al. 2019). Time intervals correspond to approximately 56 generations ago (left), approximately 31 generations ago 165 (middle), and approximately 13 generations ago (right). Light blue shows higher migration and brown shows lower migration. For the purpose of visualization, MAPS transforms the symmetric migration rate (m) into dispersal distance (*σ*) by scaling with the grid step-size (Al-Asadi et al. 2019). Dispersal distance is an “effective spatial diffusion parameter, often referred to as the ‘root mean square dispersal distance’, which can be interpreted roughly as the expected distance an individual disperses in one generation” (Al-Asadi et al. 2019). The size of each circle is proportional to the number of genetic samples.

We developed a new methodology for determining the spatial variables that best predict human migration, as inferred from shared IBD tracts. Drawing genomic data from two sources (Gurdasani et al. 2015; Scheinfeldt et al. 2019), we generated a dataset of 517,383 SNPs for 492 individuals from 35 geographic locations within our region of interest (fig. 2). After phasing the data and finding shared IBD tracts, we obtained spatial estimates of migration for three approximate time intervals (∼56, 31, and 13 generations ago) using the MAPS software (Al-Asadi et al. 2019). The migration surfaces were correlated across the time periods (Pearson *R* = 0.37–0.84) and across three independent runs (Pearson *R* = 0.54–0.97) (supplementary fig. S1, Supplementary Material online). The migration surfaces showed barriers to migration in southwestern Ethiopia and parts of Kenya and Tanzania (fig. 3). The migration surfaces were similar across the three time periods, although the most recent period, corresponding to ∼13 generations ago, has higher migration around South Sudan and lower migration in southwestern Kenya and northern Tanzania compared to the older two. The surfaces also show that the model has some bias toward inferring lower migration in areas where genomic data were collected, perhaps in part because many of these populations were targeted for their unique language or subsistence strategy, especially in Ethiopia and Tanzania. A network depicting the sharing of IBD tracts recapitulates the complex patterns of connection shown in the MAPS output (supplementary fig. S7, Supplementary Material online).

**Fig. 4.**
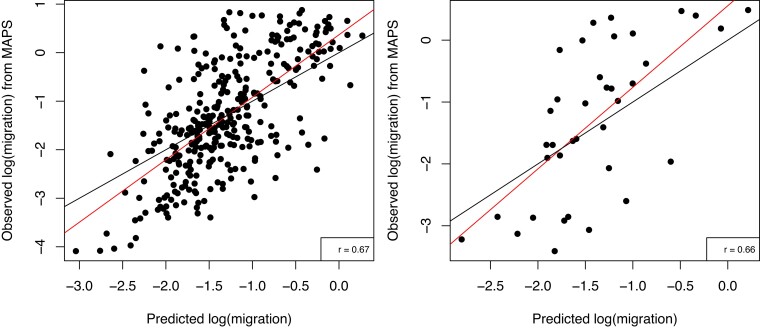
Observed migration (inferred by MAPS) versus predicted migration rate by SPRUCE for an example fold of the 10-fold cross-validation. The random forest regression was trained on 90% of the data and then used to predict migration for the same training dataset (left) and for the 10% of the data that was withheld (right). All spatial variables were included and the IBD tract lengths used were 2–4 cM (corresponding to ca. 56 generations ago). The red line is the best-fit linear regression, and the black line is *y* = *x*.

We used a random forest framework to test the effects of environment, language, and the presence of an important disease vector (tsetse fly) on migration. Specifically, we extracted the inferred migration rate value (our response variable) from 368 evenly spaced locations in our region of interest along with the values at each of the same coordinates for each of the spatial variables (our predictor variables). We performed 10-fold cross-validation, a common procedure in which a different 10% of the data is withheld each time as the testing dataset. Averaging across the ten models for each time period, the percent variance explained (RSQ) was 41.4%, 36.6%, and 46.8% for the time periods from oldest to most recent (table 1). For the majority of the folds, the correlation between predicted and actual migration was similar or even somewhat lower for the training dataset (*r*_train_) than for the test dataset (*r*_test_), indicating that a model using the full dataset would not be overfitted (fig. 4, supplementary table S4–S6, Supplementary Material online). Therefore, we created a random forest regression using the full dataset in order to produce summary results and find the most important variables (determined by the mean decrease in accuracy of the model when excluding each variable). These variables were similar across the three time periods; the minimum temperature of the coldest month and the kernel density surface were in the top four variables across the three models (table 2; supplementary table S10, Supplementary Material online). (The kernel density shows the geographic density of genetic samples and helps account for spatial autocorrelation in the model.) Other important variables included mean annual precipitation, minimum precipitation of the driest month, maximum temperature of the hottest month, elevation, and presence of the Niger-Congo languages (see supplementary figs. S2–S4 and table S2, Supplementary Material online, for more information on the predictor variables).

**Table 1.** Summary of 10-fold Cross-validation Run Across the Three Time Intervals, With and Without the Kernel Density Surface Included as a Predictor Variable.

Spatial Variables	Time Interval	Percent Variance Explained (RSQ)	*r*_train_ ± SD	*r*_test_ ± SD
All	2–4	41.4 ± 1.52	0.65 ± 0.019	0.66 ± 0.19
All	4–6	36.6 ± 0.980	0.62 ± 0.015	0.59 ± 0.14
All	6-Inf	46.8 ± 2.19	0.62 ± 0.013	0.60 ± 0.013
Excluded kernel	2–4	37.4 ± 1.52	0.62 ± 0.019	0.64 ± 0.11
Excluded kernel	4–6	30.9 ± 1.94	0.57 ± 0.016	0.59 ± 0.10
Excluded kernel	6-Inf	31.6 ± 2.00	0.57 ± 0.022	0.58 ± 0.14

Note.—For each fold, the model was trained with 90% of the data and then used to predict values for the training (90%) and test (10%) datasets. *r*_train_, correlation between observed and predicted migration for the training dataset; *r*_test_, correlation between observed and predicted migration for the testing dataset; SD, standard deviation.

**Table 2.** Summary of Results From Full Dataset Run Across the Three Time Intervals, With and Without the Kernel Density Surface Included as a Predictor Variable.

Spatial Variables	Time Interval	Percent Variance Explained (RSQ)	*r*_full_	Most important variables
All	2–4	42.3	0.66	Kernel density, minimum temperature of the coldest month, mean annual precipitation, elevation
All	4–6	38.5	0.63	Kernel density, minimum temperature of the coldest month, mean annual precipitation, precipitation of the driest month
All	6-Inf	46.6	0.68	Kernel density, presence of Niger-Congo language, minimum temperature of the coldest month, maximum temperature of the hottest month
Excluded kernel	2–4	39.3	0.64	Minimum temperature of the coldest month, mean annual precipitation, presence of Niger-Congo language, elevation
Excluded kernel	4–6	33.3	0.59	Precipitation of the driest month, elevation, minimum temperature of the driest month, presence of Niger-Congo language
Excluded kernel	6-Inf	33.1	0.58	Precipitation of the driest month, presence of Afro-Asiatic language, minimum temperature of the driest month, presence of Niger-Congo language

Note.—*r*_full_, correlation between observed and predicted migration. See for supplementary Table S3, Supplementary Material online more information on metrics.

To better understand the influence of the kernel density surface in the random forest model, we reran the 10-fold cross-validation after excluding this variable. The model performance slightly decreased; the correlation between predicted and actual migration remained >30% on average across the three time periods (supplementary table S7–S9, Supplementary Material online). The most important variables (generated with the full dataset but no kernel density) were similar to those generated without kernel density, although the presence of the Niger-Congo languages increased in importance (supplementary table S10, Supplementary Material online).

### Selection Scan

**Fig. 5.**
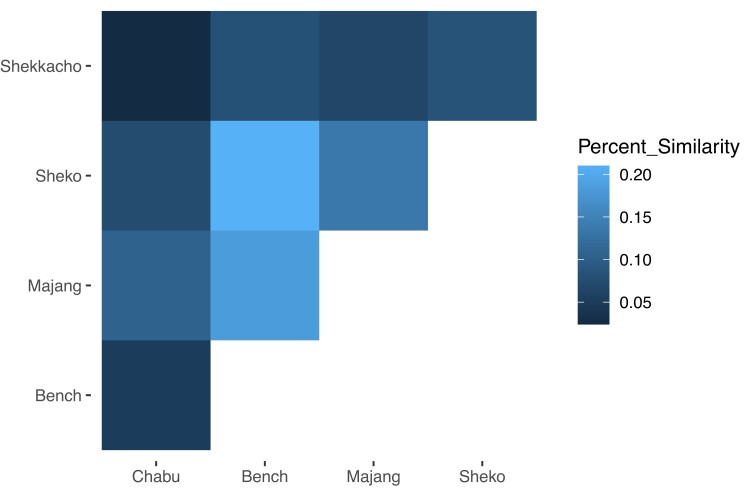
Similarity in genome-wide selection scan results between Ethiopian populations. Specifically, this table shows the percentage of overlap in top candidate genes found after filtering for the top 0.1% normalized integrated Haplotype Scores. The four agricultural groups generally shared a higher percentage of top candidate genes with each other than they did with the hunter gather group (Chabu).

Since the results from SPRUCE indicated that fine-scale environments may affect migration, we further tested whether these fine-scale differences also impact recent adaptation. We performed a genome-wide selection scan on five populations in southwestern Ethiopia who live within 100 km but derive from different ancestral groups and experience different environments (Gopalan et al. 2022) (supplementary table S11, Supplementary Material online). These population samples had high SNP density and large sample sizes, appropriate for performing a selection scan using the integrated haplotype statistic (iHS) (Voight et al. 2006, Gopalan et al. 2022). Four populations are agriculturalists, ranging from small-scale (Majang) to intensive farming (Shekkacho) (Fentaw 2007; Stauder 2007; Gopalan et al. 2022). One population, the Chabu, practiced traditional hunting and gathering until recently (Dira and Hewlett 2018; Gopalan et al. 2022). Some individuals in these populations live at moderately high elevations; for example, the Chabu live at elevations up to 2,500 m (Dira and Hewlett 2017). For the selection scan, we focused on iHS, a statistic that detects recent positive selection through the decay of ancestral and derived alleles from a query locus (Voight et al. 2006) (supplementary fig. S5, Supplementary Material online). After filtering for the 0.1% largest absolute value iHS scores, we intersected these SNPs with a list of known genes, resulting in a range of 129 (Majang) to 230 (Shekkacho) top candidate genes (supplementary Dataset S1, Supplementary Material online). Out of 664 unique genes found across the 5 populations, only 6 genes were candidate genes for all 5 populations. These six genes were related to metabolism (PTPRN2, RPH3AL), the immune system (DOCK8), cAMP binding (PRKAR1B), and vesicle-mediated transport (VPS53). We found five genes that were candidates for selection in the four agricultural populations but not in the hunter-gatherer population (the Chabu); one has a possible relationship to subsistence strategy (PASK), which regulates insulin gene expression and can play a role in Type 2 diabetes (Zhang et al. 2015). We also found that the agricultural groups shared a higher percentage of top candidate genes with each other than they did with the Chabu, with one exception (Majang was more similar to Chabu than to Shekkacho, reflecting their recent shared genetic ancestry) (fig. 5; supplementary table S12, Supplementary Material online). The Chabu and Majang also shared a common candidate gene called IRF4, which is an important regulatory transcription factor in the differentiation of immune cells. We did not find evidence of selection in genes previously associated with high elevation adaptation (EPAS1, ALPP, PTGIS, EGLN1, KCTD12, NOS2, and VDR). Interestingly, a subunit of the hypoxia-inducible factor 1 pathway (HIF1A) was detected as a candidate gene in the Chabu.

## Discussion

Here, we present a new methodology for interpreting the environmental drivers of human migration and gene flow. This method can be applied across a wide range of regions and scales and is also suitable for other organisms, given that they have a reference genome and high-resolution genetic data suitable for calling IBD tracts. We based our model on a previous method (Pless et al. 2021), which used an iterative random forest approach to infer genetic connectivity based on pairwise summary statistics (e.g., F_ST_) and the corresponding mean of environmental values between each of these pairs of populations. Our new method SPRUCE avoids the need for pairwise statistics by inferring migration rate directly through geographic space using the software MAPS (Al-Asadi et al. 2019). We applied our new method to investigate patterns of migration in eastern Africa 300–2,000 years ago and focused on different time periods by filtering for different length IBD tracts. We found a high degree of correlation in the inferred migration surfaces across the time periods, which indicates that the evolutionary forces causing IBD sharing in one time period are likely similar across proximate time periods (Al-Asadi et al. 2019). On the one hand, in some regions, we found that low migration rates corresponded to expected geographic barriers, such as the mountainous forests of southwestern Ethiopian and the arid Lake Turkana region in northern Kenya. On the other hand, some regions that we expected might be barriers (the Indian Ocean, the Ethiopian central highlands, Mount Kenya, and Mount Kilimanjaro) did not appear as barriers in the MAPS output. The lush and ecologically diverse Lake Victoria region did show evidence of promoting migration, and we observed a complex pattern of barriers and corridors in Kenya and Tanzania. For example, southeastern Kenya and northern Tanzania see strong migration between Iraqw and Kikuyu during the oldest period, potentially reflecting the incursion of agriculturalists-speaking languages from the Bantu family into the region. Then, during the intermediate ∼900-year period, we see a switch to higher migration among the Kikuyu, Taita, and Pare (all of whom speak languages in the Bantu family). Finally, most recently, there is increasing isolation among these groups.

The 25 spatial variables representing the environment (climate, elevation, land cover), tsetse fly occurrence, and language family explained ∼40% of the variance in migration rate and achieved a Pearson correlation coefficient of >0.62. Although this is lower than the *r* from our original model applied to a species of mosquito, it is on par with other landscape genetics papers that use machine learning (Murphy et al. 2010; Hether and Hoffman 2012; Fountain-Jones et al. 2017) and is noteworthy given the immense complexity of human migration and the environment in eastern Africa. Minimum temperature, mean precipitation, and elevation were consistently three of the most important variables. In some regions, these variables were correlated with each other: high-elevation regions tended to have higher precipitation and lower minimum temperatures. These conditions likely acted as a barrier to gene flow within southwestern Ethiopia and parts of southern Kenya, given the low rates of IBD sharing and inferred migration in these areas (fig. 3; supplementary fig. S7, Supplementary Material online). Many of the southern Ethiopian populations (e.g., Chabu, Aari, Hamer, Oromo, Burji, and Wolayta) cross-cut language families and subsistence strategies (Assoma 2007) and have extremely low migration rates (supplementary fig. S7, Supplementary Material online). In the most recent time period (13 generations ago), the presence/absence of the Niger-Congo languages was the second most important variable. This is likely driven by high migration areas along the southern and eastern border of our study region, which overlap with populations that speak languages in the Bantu family (supplementary fig. S3, Supplementary Material online), implying sharing this language family may promote intermarriage and migration among these groups.

Another important variable in predicting migration rates was the kernel density surface, which has higher values in areas with higher sampling density (i.e., more individuals with genetic data). We included this variable to help account for spatial autocorrelation, which arose because our genetic samples were not uniformly distributed across the region. The kernel density surface was probably important because MAPS tended to predict low migration rates near sampling locations. This may be due to neighboring populations that remain isolated from each other, while the high migration rates in-between the sampling locations were necessary to explain instances of IBD sharing across large geographic regions (supplementary fig. S7, Supplementary Material online). Interestingly, we noticed a similar bias when investigating migration barriers using the software that preceded MAPS (EEMS; Petkova et al. 2016) for southern Africa, an area that also has complex migration and isolated populations (Petkova et al. 2016; Uren et al. 2016). When we excluded the kernel density surface from our model, precipitation of the driest month and Niger-Congo language presence/absence rose in importance.

In the case of the intermediate time period model, corresponding to 31 generations ago, one of the most important variables was the occurrence of a tsetse fly group called *fusca* (forest-dwelling), and the second most import tsetse fly group was *morsitans* (savannah-dwelling) (supplementary fig. S4, Supplementary Material online). The tsetse fly is the vector of livestock trypanosomiasis and sleeping sickness in humans, and its occurrence likely limits the movement and health of pastoralist groups (Gifford-Gonzalez 2017). Cattle herding spread into Lake Turkana (northern Kenya) from Sudan by 5,000–4,000 years ago (Hildebrand and Grillo 2012) and remains a frequent subsistence strategy among many present-day pastoralists. The Kenyan and Sudanese cattle pastoralist populations occupy areas without *morsitans* (supplementary fig. S4*a*, Supplementary Material online), and many of these groups, such as the Gabra, Borana, Rendille, and Gurreh, share high cross-population IBD (supplementary fig. S7, Supplementary Material online). The *fusca* tsetse fly range is heavily represented in forested areas of southwestern Ethiopia. This may have served as a barrier to gene flow preventing Sudanese and northern Kenyan pastoralists from major incursions into the highlands.

Since the SPRUCE results indicated that fine-scale differences in the environment affect human migration, we ran a genome-wide selection scan on five neighboring populations in southwestern Ethiopia to test the effect of microenvironments on adaptation. Although these populations are proximate in geographic space, and in some cases closely related (Gopalan et al. 2022), they each show a unique pattern of positive selection using the iHS statistic (supplementary fig. S6, Supplementary Material online). We predicted that differences in pathogen exposure, subsistence strategies, and elevation would be the primary drivers of differential selection in this region, and indeed a number of the candidate genes were related to the immune response (e.g., IRF4, AXIN1, CHL1, DOCK8) or metabolism and insulin production (e.g., PNPLA2, PASK, RPH3AL, B4GALNT3). Additionally, the pairs of the agricultural groups shared more candidate genes in common than almost any of them do with the recent hunter-gatherer group (supplementary table S12, Supplementary Material online). We did not find evidence of selection for any of the specific genes previously associated with high-elevation adaptation in the literature. Two possible explanations are 1) less selection pressure due to lower elevations in Ethiopia than in the Andes or Tibetan plateau and 2) selection scans do not replicate well in African populations (Granka et al. 2012). We do highlight one candidate gene in the Chabu called HIF1A which is a part of the hypoxia-inducible factor 1 pathway. Several other HIF genes have been identified as candidate genes for adaptation to high elevation, in particular EGLN1, an O_2_ sensor that controls levels of HIF-alpha (Bigham et al. 2013; Brutsaert et al. 2019).

Like all models, SPRUCE has certain limitations and will benefit from additional work. One of the limitations is that it relies strongly on the output of another software program to predict migration rates, and the output files from this program are not intuitive to decipher (see https://github.com/evlynpless/SPRUCE-RF/blob/master/SPRUCE_Guide.txt). A pattern of complex relationships across many geographically separated individuals is inherently difficult to represent through a two-dimensional migration surface, and the migration surfaces in this study are certainly complicated and serpentine in appearance. Another option is to use IBD sharing as the predictor variable in the model, although this adds the complexity and inconvenience of using pairwise summaries of environmental variables, rather than using values corresponding to a single location.

The flexibility of SPRUCE helps overcome some of its limitations. The random forest approach can be swapped for another statistical framework, though we recommend random forest because it allows for a high level of objectivity by accepting many predictor variables, including correlated variables and both continuous and categorical variables (Breiman 2001; Hastie et al. 2009). Future extensions could also experiment with other tools to generate the response variable (migration rates). For the predictor variables (e.g., environmental data), there are a wide variety of open-source spatial rasters, or the user could generate their own spatial variables of interest including distance to a hypothesized corridor for gene flow (e.g., the coast or a river). Another improvement would be to generate spatial variables that correspond directly to the time period under investigation, for example, using historical climate reconstruction to create the climate rasters (Singarayer and Valdes 2010; Beyer et al. 2021). A current challenge to this is that historical climate reconstructions are generally performed at a coarse time scale, for example in 1,000-year intervals. Given the shallow time scale we consider in this project, we think it is reasonable to assume that the variables we included have not changed drastically in the last 2,000 years, although there have been some important changes such as a slight cooling ∼1,500 years ago followed by warming ∼1,100 years ago (Nicholson et al. 2013).

In conclusion, we present a new approach for integrating genetic and environmental data to predict the multifactorial ecological drivers of population structure and migration. In the application of SPRUCE to a complex dataset in eastern Africa, we find that recent fine-scale human population structure is predicted by precipitation, minimum temperature, elevation, and presence of a vector for trypanosomiasis. Our method is complementary to efforts utilizing historical climate reconstructions to reconstruct human migration corridors (e.g., Beyer et al. 2021) and could now be applied to a variety of regions to infer which environmental variables are “limiting factors” for migration, depending on the ecological and temporal context. The rapid increase in dense genetic datasets for many species will further facilitate the utility of this approach.

## Materials and Methods

### SPRUCE Model

We provide the full scripting procedure and additional commentary on SPRUCE here: https://github.com/evlynpless/SPRUCE-RF. MAPS software and resources can be found here: https://github.com/halasadi/MAPS.

#### Environmental and spatial data

Climate and landcover data were downloaded from free, online repositories and were edited and cropped using Geospatial Data Abstraction Library (GDAL/OGR Contributors 2021) under the Bash environment. Most datasets were available at 1-km resolution; otherwise, they were resampled to a pixel size of 1 km^2^. Elevation was derived from MERIT DEM (Multi-Error-Removed Improved Terrain Digital Elevation Models) (Yamazaki et al. 2017), and slope was derived from the Geomorpho90m dataset (Amatulli et al. 2020). Mean annual temperature, maximum temperature of the hottest month, minimum temperature of the coldest month, mean precipitation of the wettest month, and mean precipitation of the driest month were obtained from CHELSA (climatologies at high resolution for the earth's land surface areas) (Karger et al. 2017). We also included aridity from the Global Aridity Index, and gross primary production, a measure of vegetative photosynthesis (Zomer et al. 2007, 2008). Each landcover type was represented by a separate raster, in which values from 0 to 1 indicated what percent of that pixel was covered with each landcover type (Tuanmu and Jetz 2014).

Tsetse suitability maps for the three main groups of flies (*morsitans*, *fusca*, and *palpalis*) were downloaded as shapefiles from the Food and Agriculture Organization of the United Nations (Cecchi et al. 2008) and converted to rasters using qGIS 2.18. Language data (including language family and geographic coordinates) were downloaded from Ethiolang and Wikitongues and categorized by language family using R Statistical Software (v.4.0.2; R Core Team 2020). Local convex hulls were created for the three primary language families in eastern Africa, Afro-Asiatic, Nilo-Saharan, and Niger-Congo, using the R package “LoCoH” (supplementary fig. S3, Supplementary Material online) (Getz et al. 2007). To address spatial autocorrelation among the geographic coordinates of the individuals, we included a kernel density raster (bandwidth 200 km) created using the R package “KernSmooth” (Wand et al. 2015).

The values at each deme for each spatial variable were extracted with the R package rgdal (“gdalinlocationinfo”) (Bivand et al. 2021), with the exception of the language maps which were extracted using the R package sf (“st_contains”) (Pebesma 2018). The location and correct order of the demes to match the inferred migration rates were found in the demes.txt output file from the MAPS software.

#### Genomic Data, Phasing, and IBD

For the SPRUCE analysis, we included dense SNP array data from 35 populations in eastern Africa from two sources (Gurdasani et al. 2015; Scheinfeldt et al. 2019) (supplementary table S1, Supplementary Material online). These populations were primarily from Ethiopia, Kenya, and Tanzania and include ten populations that practice or recently practiced hunting and gathering as their primary subsistence strategy. In preparation for merging the two datasets, all variants were oriented to match the 1000 Genomes reference using a custom script. We flipped SNPs that were on the wrong strand, and we excluded SNPs that did not match the 1000 Genomes reference. We merged the datasets using plink1.9 (−merge-mode 1) and removed loci with genotype missingness >95% (Purcell et al. 2007; Purcell 2016). The merged dataset included 517,383 SNPs and 821 individuals from 50 populations. We performed principal component analysis with Plink, which confirmed the relationships among the populations were as expected. In preparation for calling IBD segments, we performed phasing with SHAPEIT2 using a reference panel of phased individuals from the 1000 Genomes project Phase 3 dataset, the –duohmm option, and a window size of 5 Mb (Delaneau et al. 2013). We then removed first- and second-degree relatives identified by KING (Manichaikul et al. 2010). Shared IBD segments were obtained with hap-ibd v1.0 (Zhou et al. 2020) and repaired with the program merge-ibd-segments. We excluded 15 populations that fell outside our geographic region of interest (top left = 12.5°N, 30.0°E, bottom right = -7.0°S, 44.0°E), leaving 35 populations and 492 individuals for the SPRUCE analysis (fig. 2).

To visualize patterns of IBD sharing, we created networks in Cytoscape 3.9.1. (Shannon et al. 2003). In these networks, each individual is a node, and the locations of the nodes roughly correspond to geographic positions. The edges represent the number of shared IBD segments, and for ease of interpretation, we focused on IBD segments >6 cM (corresponding to the most recent time interval considered in MAPS).

#### Population Names and Coordinates

Throughout the manuscript, we use population names from the original sources with the exception of the “Sabue,” which we refer to as “Chabu” (Dira and Hewlett 2017). Likewise, we use geographic coordinates from the original publications when available. Coordinates that were not included in the original sources (Pagani et al. 2012; Gurdasani et al. 2015) were taken from Gopalan et al. (2022). Coordinates not included in Scheinfeldt et al. (2019) were inferred from a map in the manuscript using a custom script.

#### MAPS

In preparation for running MAPS, we adapted and ran code provided by the program authors to convert the output of hap-ibd into a matrix format read by MAPS, and in the process, we filtered IBD calls into three categories: 2–4, 4–6, and >6 cM. Under a simplistic model of infinite population size, the mean ages of the categories correspond to 56.25, 31.25, and 12.50 generations, respectively (specifically, the expected coalescent time [in generations]) of IBD segments between length L_1_ and L_2_ cM is approximately (300/4)*((1/L_1_) + (1/L_2_)) if the effective population size is sufficiently large (see S1 Appendix of Al-Asadi et al. 2019). Choosing the number of demes is a trade-off between finding higher-resolution migration patterns and a sharp increase in computational time, and we ultimately chose 400 demes. We averaged inferred migration over three replicates to mediate any outlier results. The number of MCMC iterations in each replicate was set to 5 million, the burn-in was set to 2 million, and we thinned every 2,000 iterations as in Al-Asadi et al. (2019). Migration rate “m” is the average scaled rate into a given deme over the bidirectional migration occurring along all 6 edges in the lattice. Migration surfaces were visualized using the R package “plotmaps” (Al-Asadi 2017). For visualization without grid lines, we also report dispersal distance, which is the effective spatial diffusion parameter, calculated by scaling migration by the step size of the grid used by the MAPS software (Al-Asadi et al. 2019). Dispersal distance is roughly equal to the expected distance an migrant disperses in one generation (Al-Asadi et al. 2019).

#### SPRUCE

We calculated the response variable, migration rate at each deme, by taking values in the “mRates.txt” output file from MAPS and converting them to 10^m^ (Al-Asadi, personal communication). Since migration rates were unbalanced among populations and skewed toward low values, we used a log transformation before including them in the model (supplementary fig. S8, Supplementary Material online). The predictor variables were the values of 25 spatial variables related to climate, ecology, disease vectors, and language at each deme, as well as the kernel density surface to account for spatial autocorrelation (supplementary table S2, Supplementary Material online). All variables were numeric and continuous except for language which was coded as “1” for the language family present, and “0” for the language family not present. We performed random forest regressions using the R package randomForestSRC 2.9.3 (Ishwaran et al. 2022). We used the “tune” function to find the optimal number of features to consider at each split point (“mtry”) and the minimum size of terminal nodes (“nodesize”) based on out-of-bag error. We performed random forest regressions for each of the three time intervals of interest (56.25, 31.25, and 12.50 generations ago). We ran the analyses with and without including the kernel density surface as a predictor variable to better understand its influence on the model. In addition to creating models with the full dataset, we also performed 10-fold cross-validation. In other words, we used 90% of the dataset for training and 10% for testing, and we repeated this procedure 10 times, reserving a different 10% of the data for testing each time.

### Genome-wide Selection Scans

#### Genomic Data

Although the SPRUCE dataset described above gave us the broadest possible geographic spread in eastern Africa, some populations had relatively low sample sizes. We, therefore, considered an alternative high SNP density, high sample size dataset of five Ethiopian populations previously published in (Gopalan et al. 2022) and available in dbGaP at phs001123.v2.p2 (supplementary table S11, Supplementary Material online). We tested for selection in these five populations from southwestern Ethiopia: Bench, Sheko, Majang, Shekkacho, and Chabu. Fifty individuals from each population (and 88 from the Chabu) were genotyped on the Illumina Infinium MultiEthnic Global Array, which assays over 1.7 million genetic markers. Gopalan et al. (2022) filtered SNPs to remove samples with call rates <90%. Variants with minor allele frequency <5% were updated with zCall genotypes, and then variants were removed if they had >15% missing data, heterozygosity >= to 80%, or cluster separation <= to 2%.

#### Selection Scan

To prepare genetic data for haplotype-based selection scans, we implemented phasing using a reference panel of phased individuals from the 1000 Genomes project Phase 3 dataset (The 1000 Genomes Project Consortium 2015), the –duohmm option, and a window size of 5 Mb in SHAPEIT2 (Delaneau et al. 2013). PONDEROSA (Williams et al. 2020), an algorithm for accurately recovering family-level relationships from genome-wide data, was used to exclude individuals with a second-degree or closer relative in the dataset, leaving 222 individuals. For the selection scan, we used integrated Haplotype Score (iHS) (Voight et al. 2006), a metric based on extended haplotype homozygosity (EHH) (Sabeti et al. 2002) which measures the decay of identity of haplotypes, as a function of distance, from a specified “core” allele. Calculating iHS involves taking the integral of EHH. We calculated iHS using selscan 1.3.0 (Szpiech and Hernandez 2014) and default parameters and a genetic map based on crossovers in 30,000 unrelated African Americans (Hinch et al. 2011). We found the absolute value of the normalized iHS scores and filtered for the 0.1% largest absolute value iHS scores. We annotated these genetic positions with a gene range list provided by Plink (https://www.cog-genomics.org/plink/1.9/resources).

To compare top gene hits across the five populations, we created Venn Diagrams using InteractiVenn (Heberle et al. 2015). We generated plots showing iHS scores across the genome using the R package “rehh” (Gautier and Vitalis 2012).

## Supplementary Material

msad094_Supplementary_Data

## Data Availability

No new data were generated or analyzed in support of this research. Publicly available datasets can be accessed at dbGaP accessions phs001780.v1.p1, phs001123.v2.p2, and EGA accession EGAD00010001221. The software developed for this manuscript is available at https://github.com/evlynpless/SPRUCE-RF.
