# Supplementary material for: Predicting Environmental and Ecological Drivers of Human Population Structure
Source: Mol Biol Evol. 2023 May 5;40(5):msad094. doi: 10.1093/molbev/msad094 (PMC10172848; doi:10.1093/molbev/msad094)
Supplement: msad094_Supplementary_Data [file msad094_supplementary_data.zip › MBE_SI_revision.pdf]

Supplementary Materials for

Predicting environmental and ecological drivers of human population structure

Evlyn Pless[1*], Anders M. Eckburg[1], Brenna M. Henn [1,2*]

[1]Department of Anthropology, University of California, Davis
[2]UC Davis Genome Center, University of California, Davis
*Corresponding authors

The supplementary materials include:

Tables S1 to S12
Figures S1 to S7
Legend for Dataset S1
SI References

Other supplementary materials for this manuscript:

Dataset S1

Table S1. Populations included in SPRUCE model. Number (consistent with Fig. 1), population name, country, language family, traditional subsistence strategy, longitude, latitude, number of individuals, and reference: [1] is Scheinfeldt et al. 2019, [2] is Gurdasani et al. 2015 (originally collected for Pagani et al. 2012).

Number	Population	Country	Language	Subsistence	Longitude	Latitude	N	Reference
1	Aari	Ethiopia	Afro-Asiatic	Agropastoralist	36.6106	6.4108	10	[1]
2	Amhara	Ethiopia	Afro-Asiatic	Agriculturalist and/or pastoralist	39	10	25	[2]
3	Boni	Kenya	Afro-Asiatic	Hunter-Gatherer	40.6811	-0.7333	15	[1]
4	Borana	Kenya	Afro-Asiatic	Pastoralist	39.2069	2.9284	18	[1]
5	Burji	Kenya	Afro-Asiatic	Agriculturalist	38.1608	5.4487	14	[1]
6	Chabu	Ethiopia	Nilo-Saharan / Isolate	Hunter-Gatherer	35.3239	7.4402	8	[1]
7	Dahalo	Kenya	Afro-Asiatic	Hunter-Gatherer	40.3482	-1.9221	9	[1]
8	Datog	Tanzania	Nilo-Saharan	Pastoralist	35.42	-4.21	16	[1]
9	Dinka	Sudan	Nilo-Saharan	Pastoralist	31.5924	6.0891	12	[1]
10	Elmolo	Kenya	Afro-Asiatic	Hunter-Gatherer	36.4488	2.8333	6	[1]
11	Ethiopian Somali	Ethiopia	Afro-Asiatic	Agriculturalist and/or pastoralist	42	9	24	[2]
12	Gabra	Kenya	Afro-Asiatic	Pastoralist	37.6852	2.9759	9	[1]
13	Gumuz	Ethiopia	Isolate	Horticulturalist	35.9	11	22	[2]
14	Gurreh	Kenya	Afro-Asiatic	Pastoralist	34.832	3.1661	7	[1]
15	Hadza	Tanzania	Khoisan	Hunter-Gatherer	33.14	-3.33	16	[1]
16	Hamer	Ethiopia	Afro-Asiatic	Pastoralist	36.5463	5.3814	10	[1]
17	Ilchamus	Kenya	Nilo-Saharan	Pastoralist	36.4964	1.1213	16	[1]
18	Iraqw	Tanzania	Afro-Asiatic	Agriculturalist	35.48	-3.5	22	[1]
19	Kikuyu	Kenya	Niger-Kordofanian	Agriculturalist	37.2572	-1.3515	14	[1]
20	Luo	Kenya	Nilo-Saharan	Pastoralist	34.4516	-0.5906	31	[1]
21	Ogiek	Kenya	Nilo-Saharan	Hunter-Gatherer	35.9733	-0.4004	12	[1]
22	Orma	Kenya	Afro-Asiatic	Pastoralist	39.8727	-2.9683	14	[1]
23	Oromo	Ethiopia	Afro-Asiatic	Agropastoralism	37	8	20	[2]
24	Pare	Kenya	Niger-Kordofanian	Agriculturalist	37.8754	-4.7753	12	[1]
25	Pokot	Kenya	Nilo-Saharan	Pastoralist	35.2124	2.0249	5	[1]
26	Rangi	Tanzania	Niger-Kordofanian	Agriculturalist	35.79	-4.91	14	[1]
27	Rendille	Kenya	Afro-Asiatic	Pastoralist	37.6377	1.9773	14	[1]
28	Sandawe	Tanzania	Khoisan	Hunter-Gatherer	34.88	-5.24	24	[1]
29	Sengwer	Kenya	Nilo-Saharan	Hunter-Gatherer	34.5467	0.7409	12	[1]
30	Taita	Kenya	Niger-Kordofanian	Agropastoralist	38.3034	-3.2061	7	[1]
31	Taveta	Kenya	Niger-Kordofanian	Agropastoralist	38.3034	-3.2061	2	[1]
32	Tugen	Kenya	Nilo-Saharan	Pastoralist	35.3551	0.9311	15	[1]
33	Wata	Kenya	Afro-Asiatic	Hunter-Gatherer	37.0195	3.927	4	[1]
34	Wolayta	Ethiopia	Afro-Asiatic	Agropastoralist	37	6	23	[2]
35	Yaaku	Kenya	Afro-Asiatic	Hunter-Gatherer	37.2097	0.408	10	[1]

Table S2. List of spatial variables included as predictor variables in the SPRUCE model. Abbreviation, description, source, resolution of the original dataset and resampling method used (if any).

Abbreviation	Description	Source name	Original resolution
Aridity	Global Aridity Index	CIGAR CSI	$1km^2$
MeanTemp	Annual mean temperature	CHELSA climate data	$1km^2$
MaxTemp	Maximum temperature of the warmest month	CHELSA climate data	$1km^2$
MinTemp	Coldest temperature of the coldest month	CHELSA climate data	$1km^2$
MeanPrec	Annual precipitation	CHELSA climate data	$1km^2$
PrecWet	Precipitation of the wettest month	CHELSA climate data	$1km^2$
PrecDry	Precipitation of the driest month	CHELSA climate data	$1km^2$
Evergreen	Evergreen/deciduous needleleaf trees (%)	Tuanmu and Jetz 2014	$1km^2$
Decid	Deciduous broadleaf trees (%)	Tuanmu and Jetz 2014	$1km^2$
Tree	Mixed/other trees (%)	Tuanmu and Jetz 2014	$1km^2$
Shrub	Shrubs (%)	Tuanmu and Jetz 2014	$1km^2$
Herb	Herbaceous vegetation (%)	Tuanmu and Jetz 2014	$1km^2$
Crop	Cultivated and managed vegetation (%)	Tuanmu and Jetz 2014	$1km^2$
Barren	Barren including spare shrub/herbaceous cover (%)	Tuanmu and Jetz 2014	$1km^2$
Water	Open water (%)	Tuanmu and Jetz 2014	$1km^2$
Slope	Slope	Amatulli *et al.* 2020	$90m^2$, resampled by taking mean of pixels in $1km^2$
Elevation	Elevation	MERIT DEM	$90m^2$, resampled by taking mean of pixels in $1km^2$
GPP	Gross primary production, a measure of vegetation photosynthesis	Zhang *et al.* 2017	$500m^2$, bilinear resampling
Fusc	Predicted area of suitability for tsetse flies (fusca): vector of livestock trypanosomiasis	Cecchi 2002	5km, not resampled
Mors	Predicted area of suitability for tsetse flies (morsitans): primary vector of T. rhodesiense in east Africa (mostly infect wildlife)	Cecchi 2002	5km, not resampled
Palp	Predicted area of suitability for tsetse flies (palpalis): primary vector of T. gambiense in west and central Africa	Cecchi 2002	5km, not resampled
LangAA	Inferred distribution of Afroasiastic languages	Glottolog and Wikitongues	NA (vector file)
LangNC	Inferred distribution of Niger-Congo languages	Glottolog and Wikitongues	NA (vector file)
LangNS	Inferred distribution of Nilo-Saharan languages	Glottolog and Wikitongues	NA (vector file)
Kernel	Kernel density map of sampled sites (bandwidth = 200km)	NA	$1km^2$

Table S3. Description and equations for each performance metric recorded for the model. The equations reference the randomforestSRC package in R and variables are defined as follows: RF = Random Forest model under consideration, M = migration rate (inferred by MAPS), TestingData = 10% of the dataset, TrainingData = 90% of the dataset.

**Metrics for 10-fold cross-validation / training and validation models**		
**Abbreviation**	**Description**	**Equation**
$RMSE_{train}$	Root mean square error of model for training dataset	sqrt(mean((RF$predicted.oob - TrainingData$M)^2))
$RMSE_{test}$	Root mean square error of model for validation dataset	sqrt(mean((predict.rfsrc(RF, TestingData)$predicted - TestingData$M)^2))
r_{train}	Pearson correlation between predicted and observed genetic distance for training dataset	cor(RF$predicted.oob,TrainingData$M)
r_{test}	Pearson correlation between predicted and observed genetic distance for validation dataset	cor((predict.rfsrc(RF, TestingData))$predicted, TestingData$M)
**Metrics for full dataset run**		
**Abbreviation**	**Description**	**Equation**
RSQ_{full}	Psuedo R-squared (percent variance explained by model, calculated by randomForesSRC)	1 - mse / var(y)
$RMSE_{full}$	Root mean square error of model	sqrt(mean((RF$predicted.oob - FullData$M)^2))
r_{full}	Pearson correlation between predicted and observed genetic distance for full dataset	cor(RF$predicted.oob, FullData$M)

Table S4: Results for 10-fold cross-validation for IBD tracts of 2-4cM with all spatial variables included. For each fold, the model was trained with 90% of the data and then used to predict values for the training (90%) and test (10%) datasets. $\mathbf{r_{train}}$ = correlation between observed and predicted migration for the training dataset; $\mathbf{r_{test}}$ = correlation between observed and predicted migration for the testing dataset; $\mathbf{RMSE_{train}}$ = root mean squared error of model for training dataset; $\mathbf{RMSE_{test}}$ = root mean squared error of model for testing dataset. For detailed information about these metrics, see Table S3.

**Spatial variables**	**IBD cM**	**Fold**	$\mathbf{R_{train}}$	$\mathbf{R_{test}}$	$\mathbf{RMSE_{train}}$	$\mathbf{RMSE_{test}}$	**RSQ**
All	2-4	1	0.66	0.62	0.88	0.85	42.57
All	2-4	2	0.65	0.66	0.87	1.02	40.64
All	2-4	3	0.66	0.63	0.87	0.86	42.74
All	2-4	4	0.62	0.71	0.91	0.77	38.70
All	2-4	5	0.64	0.76	0.89	0.81	40.23
All	2-4	6	0.65	0.64	0.87	1.00	41.64
All	2-4	7	0.65	0.79	0.89	0.86	40.27
All	2-4	8	0.64	0.77	0.90	0.68	40.86
All	2-4	9	0.67	0.52	0.88	0.84	43.64
All	2-4	10	0.67	0.66	0.87	0.93	42.76
All	2-4	mean	0.65	0.68	0.88	0.86	41.41
All	2-4	stdev	0.01	0.08	0.01	0.10	1.52

Table S5: Results for 10-fold cross-validation for IBD tracts of 4-6cM with all spatial variables. For each fold, the model was trained with 90% of the data and then used to predict values for the training (90%) and test (10%) datasets. $\mathbf{r_{train}}$ = correlation between observed and predicted migration for the training dataset; $\mathbf{r_{test}}$ = correlation between observed and predicted migration for the testing dataset; $\mathbf{RMSE_{train}}$ = root mean squared error of model for training dataset; $\mathbf{RMSE_{test}}$ = root mean squared error of model for testing dataset. For detailed information about these metrics, see Table S3.

**Spatial variables**	**IBD cM**	**Fold**	$\mathbf{R_{train}}$	$\mathbf{R_{test}}$	$\mathbf{RMSE_{train}}$	$\mathbf{RMSE_{test}}$	**RSQ**
All	4-6	1	0.62	0.73	0.95	0.90	36.95
All	4-6	2	0.60	0.58	0.95	1.07	36.00
All	4-6	3	0.61	0.51	0.95	1.07	36.84
All	4-6	4	0.61	0.54	0.95	1.01	37.41
All	4-6	5	0.60	0.68	0.98	0.85	34.78
All	4-6	6	0.59	0.68	0.97	0.89	35.21
All	4-6	7	0.62	0.68	0.96	0.84	37.44
All	4-6	8	0.62	0.62	0.95	0.94	37.75
All	4-6	9	0.62	0.70	0.95	0.89	37.08
All	4-6	10	0.62	0.62	0.96	0.91	36.79
All	4-6	mean	0.61	0.63	0.96	0.94	36.63
All	4-6	stdev	0.01	0.07	0.01	0.09	0.98

Table S6: Results for 10-fold cross-validation for IBD tracts >6cM with all spatial variables. For each fold, the model was trained with 90% of the data and then used to predict values for the training (90%) and test (10%) datasets. $\mathbf{r_{train}}$ = correlation between observed and predicted migration for the training dataset; $\mathbf{r_{test}}$ = correlation between observed and predicted migration for the testing dataset; $\mathbf{RMSE_{train}}$ = root mean squared error of model for training dataset; $\mathbf{RMSE_{test}}$ = root mean squared error of model for testing dataset. For detailed information about these metrics, see Table S3.

**Spatial variables**	**IBD cM**	**Fold**	$\mathbf{R_{train}}$	$\mathbf{R_{test}}$	$\mathbf{RMSE_{train}}$	$\mathbf{RMSE_{test}}$	**RSQ**
All	6-Inf	1	0.68	0.80	0.94	0.92	45.81
All	6-Inf	2	0.69	0.53	0.94	1.06	47.38
All	6-Inf	3	0.71	0.52	0.91	1.14	49.44
All	6-Inf	4	0.67	0.76	0.97	0.83	44.31
All	6-Inf	5	0.66	0.83	0.97	0.79	43.12
All	6-Inf	6	0.70	0.71	0.93	0.90	48.53
All	6-Inf	7	0.69	0.51	0.95	0.96	47.62
All	6-Inf	8	0.71	0.53	0.93	0.95	49.78
All	6-Inf	9	0.68	0.83	0.95	0.87	45.21
All	6-Inf	10	0.68	0.70	0.94	0.97	46.55
All	6-Inf	mean	0.69	0.67	0.94	0.94	46.78
All	6-Inf	stdev	0.02	0.14	0.02	0.11	2.19

Table S7: Results for 10-fold cross-validation for IBD tracts of 2-4cM with kernel density surface excluded. For each fold, the model was trained with 90% of the data and then used to predict values for the training (90%) and test (10%) datasets. $\mathbf{r_{train}}$ = correlation between observed and predicted migration for the training dataset; $\mathbf{r_{test}}$ = correlation between observed and predicted migration for the testing dataset; $\mathbf{RMSE_{train}}$ = root mean squared error of model for training dataset; $\mathbf{RMSE_{test}}$ = root mean squared error of model for testing dataset. For detailed information about these metrics, see Table S3.

**Spatial variables**	**IBD cM**	**Fold**	$\mathbf{R_{train}}$	$\mathbf{R_{test}}$	$\mathbf{RMSE_{train}}$	$\mathbf{RMSE_{test}}$	**RSQ**
No kernel	2-4	1	0.62	0.67	0.92	0.78	37.13
No kernel	2-4	2	0.63	0.62	0.91	0.92	37.87
No kernel	2-4	3	0.64	0.44	0.90	0.96	39.68
No kernel	2-4	4	0.61	0.68	0.92	0.88	35.93
No kernel	2-4	5	0.63	0.62	0.92	0.68	39.14
No kernel	2-4	6	0.60	0.63	0.92	1.05	35.26
No kernel	2-4	7	0.60	0.59	0.91	1.08	35.50
No kernel	2-4	8	0.63	0.64	0.90	0.90	38.58
No kernel	2-4	9	0.62	0.66	0.90	0.91	37.89
No kernel	2-4	10	0.62	0.60	0.91	0.95	36.85
No kernel	2-4	mean	0.62	0.62	0.91	0.91	37.38
No kernel	2-4	stdev	0.01	0.07	0.01	0.12	1.52

Table S8: Results for 10-fold cross-validation for IBD tracts of 4-6cM with kernel density surface excluded. For each fold, the model was trained with 90% of the data and then used to predict values for the training (90%) and test (10%) datasets. $\mathbf{r_{train}}$ = correlation between observed and predicted migration for the training dataset; $\mathbf{r_{test}}$ = correlation between observed and predicted migration for the testing dataset; $\mathbf{RMSE_{train}}$ = root mean squared error of model for training dataset; $\mathbf{RMSE_{test}}$ = root mean squared error of model for testing dataset. For detailed information about these metrics, see Table S3.

**Spatial variables**	**IBD cM**	**Fold**	$\mathbf{R_{train}}$	$\mathbf{R_{test}}$	$\mathbf{RMSE_{train}}$	$\mathbf{RMSE_{test}}$	**RSQ**
No kernel	4-6	1	0.60	0.30	0.98	1.08	34.81
No kernel	4-6	2	0.54	0.68	1.01	0.99	28.11
No kernel	4-6	3	0.56	0.50	1.00	1.14	30.52
No kernel	4-6	4	0.58	0.47	1.01	0.93	31.85
No kernel	4-6	5	0.56	0.52	1.00	1.10	29.79
No kernel	4-6	6	0.56	0.60	1.01	1.01	29.39
No kernel	4-6	7	0.58	0.53	1.00	0.98	32.42
No kernel	4-6	8	0.59	0.61	0.99	0.98	32.41
No kernel	4-6	9	0.57	0.72	1.01	0.86	30.18
No kernel	4-6	10	0.55	0.80	1.01	0.79	29.83
No kernel	4-6	mean	0.57	0.57	1.00	0.99	30.93
No kernel	4-6	stdev	0.02	0.14	0.01	0.11	1.94

Table S9: Results for 10-fold cross-validation for IBD tracts >6cM with kernel density surface excluded. For each fold, the model was trained with 90% of the data and then used to predict values for the training (90%) and test (10%) datasets. $\mathbf{r_{train}}$ = correlation between observed and predicted migration for the training dataset; $\mathbf{r_{test}}$ = correlation between observed and predicted migration for the testing dataset; $\mathbf{RMSE_{train}}$ = root mean squared error of model for training dataset; $\mathbf{RMSE_{test}}$ = root mean squared error of model for testing dataset. For detailed information about these metrics, see Table S3.

**Spatial variables**	**IBD cM**	**Fold**	$\mathbf{R_{train}}$	$\mathbf{R_{test}}$	$\mathbf{RMSE_{train}}$	$\mathbf{RMSE_{test}}$	**RSQ**
No kernel	6-Inf	1	0.57	0.60	1.06	1.09	32.06
No kernel	6-Inf	2	0.59	0.52	1.06	1.08	32.67
No kernel	6-Inf	3	0.59	0.47	1.05	1.09	34.06
No kernel	6-Inf	4	0.54	0.55	1.08	1.10	29.22
No kernel	6-Inf	5	0.58	0.63	1.05	1.09	32.79
No kernel	6-Inf	6	0.60	0.34	1.07	0.96	33.63
No kernel	6-Inf	7	0.55	0.69	1.09	0.88	29.77
No kernel	6-Inf	8	0.54	0.71	1.07	1.13	28.75
No kernel	6-Inf	9	0.58	0.47	1.06	1.13	33.28
No kernel	6-Inf	10	0.55	0.64	1.07	1.07	29.79
No kernel	6-Inf	mean	0.57	0.56	1.07	1.06	31.60
No kernel	6-Inf	stdev	0.02	0.11	0.01	0.08	2.00

Table S10. Results for full dataset run. **RSQ_{full}** = Pseudo R-squared (percent variance explained by the model); **r_{full}** = correlation between observed and predicted migration; **$RMSE_{full}$** = root mean squared error of model. **Most important variables** are the four most important variables. For detailed information about these metrics, see Table S3.

**Spatial variables**	**IBD cM**	**RSQ_{full}**	**r_{full}**	**$RMSE_{full}$**	**Most important variables**
All	2-4	42.3	0.664	2.02	Kernel, MinTemp, MeanPrec, Elevation
All	4-6	38.5	0.626	2.18	Kernel, MinTemp, MeanPrec, PrecDry
All	6-Inf	46.6	0.682	2.17	Kernel, LangNC, MinTemp, MaxTemp
Excluded kernel	2-4	39.3	0.635	2.07	MinTemp, MeanPrec, LangNC, Elevation
Excluded kernel	4-6	33.3	0.591	2.27	PrecDry, Elevation, MinTemp, LangNC
Excluded kernel	6-Inf	33.1	0.578	2.43	PrecDry, LangAA, MinTemp, LangNC

Table S11. Populations from southwestern Ethiopia included in the selection scan analysis. Population name, longitude, latitude, number of individuals, current or recent subsistence strategy, language family, and reference.

Population	Longitude	Latitude	N	Subsistence	Language	Reference
Bench	35.6	7	48	Agriculture	Afro-Asiatic	Gopalan et al. 2021
Chabu	35.2	7.5	83	Hunter-Gatherer	Nilo-Saharan / Isolate	Gopalan et al. 2021
Majang	35.3	7.2	49	Agriculture	Nilo-Saharan	Gopalan et al. 2021
Shekkacho	35.4	7.2	46	Agriculture	Afro-Asiatic	Gopalan et al. 2021
Sheko	35.5	7	50	Agriculture	Afro-Asiatic	Gopalan et al. 2021

Table S12: Similarity in genome-wide selection scan results between Ethiopian populations. Specifically, this table shows the percentage of overlap in top candidate genes found using normalized integrated Haplotype Scores (iHS). The agricultural groups shared a higher percentage of top candidate genes with each other than they did with the hunter gather group (Chabu), with one exception shown in bold.

	**Chabu**	**Bench**	**Majang**	**Sheko**	**Shekkacho**
**Chabu**		5%	10.7%	7.4%	2.4%
**Bench**	5%		18.3%	21%	8.1%
**Majang**	10.7%	18.3%		13.6%	**6.5%**
**Sheko**	7.4%	21%	13.6%		8.4%
**Shekkacho**	2.4%	8.1%	**6.5%**	8.4%	

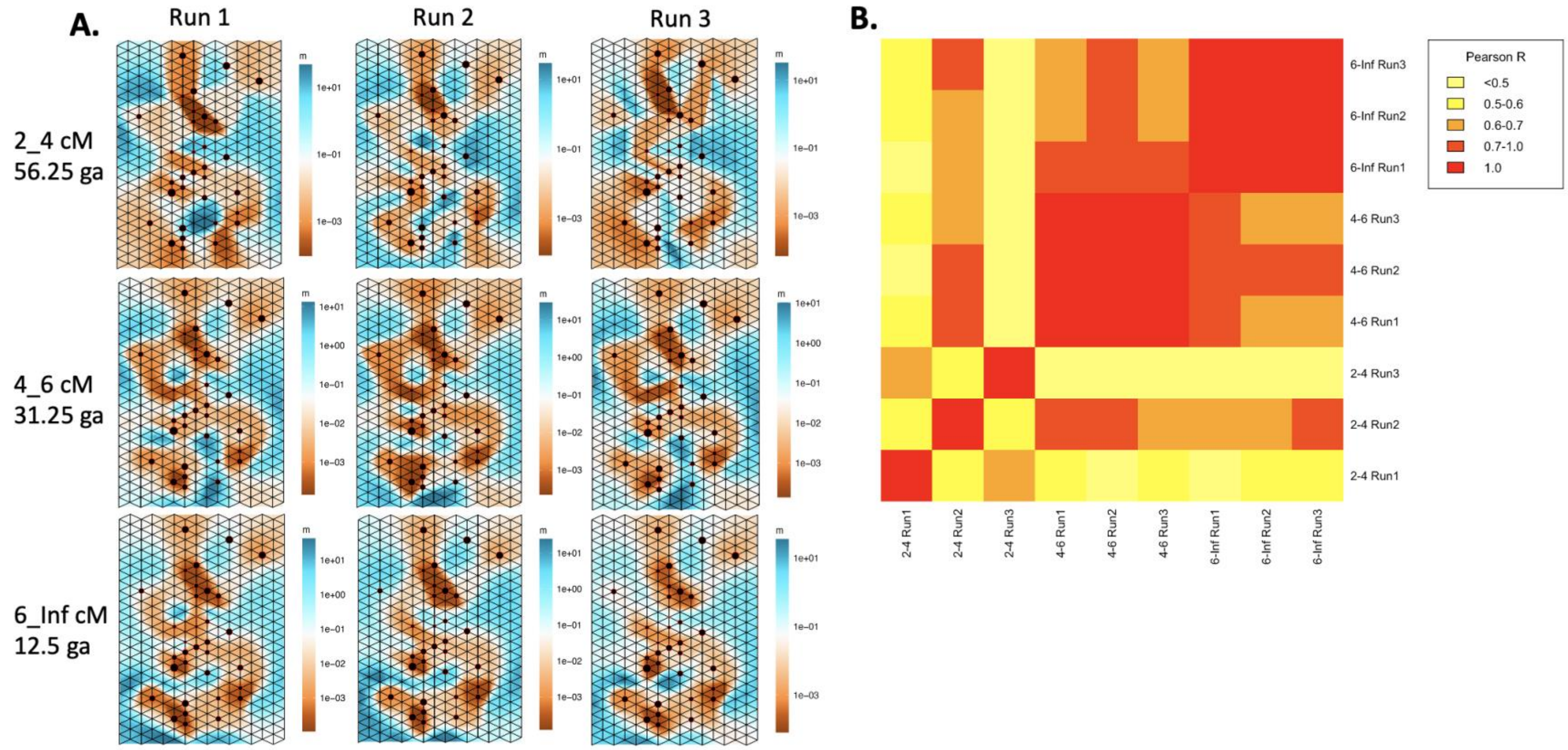

Figure S1: Migration surfaces generated by plotmaps (Al-Asadi et al. 2017), showing high correlation across runs and across time periods. Each column shows an independent run of the MAPS software (Al-Asadi et al. 2019), and each row represents a different time period in ga (generations ago) (i.e. different length identical by descent (IBD) tracts measured in cM (centimorgans)).

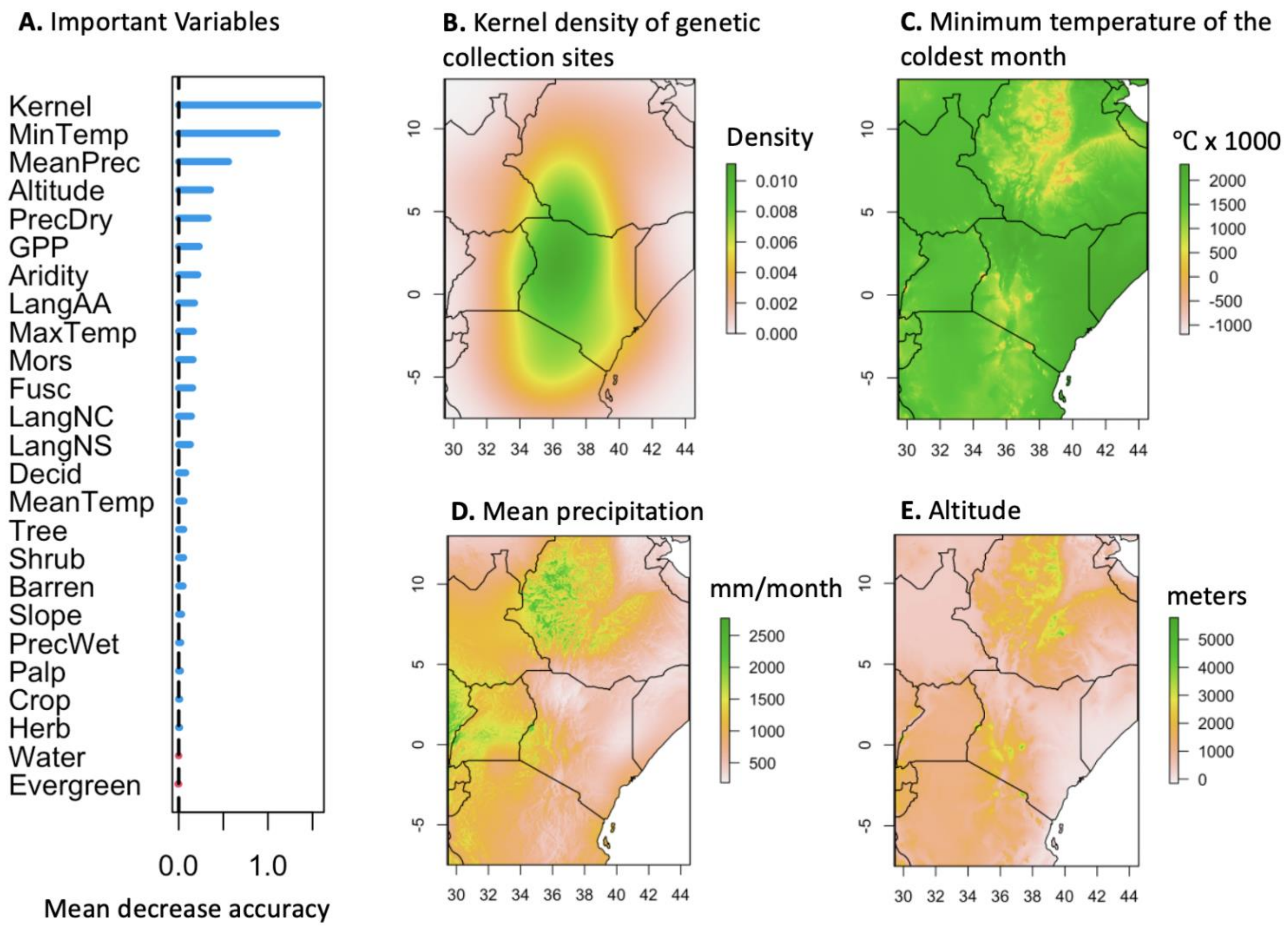

Figure S2: A. Most important variables in the SPRUCE random forest model for 2-4cM IBD tracts (~56 generations ago), determined by mean decrease in accuracy of the model when excluding each variable. B-E. Rasters of the four most important variables from this model run, overlaid with the country lines from eastern Africa.

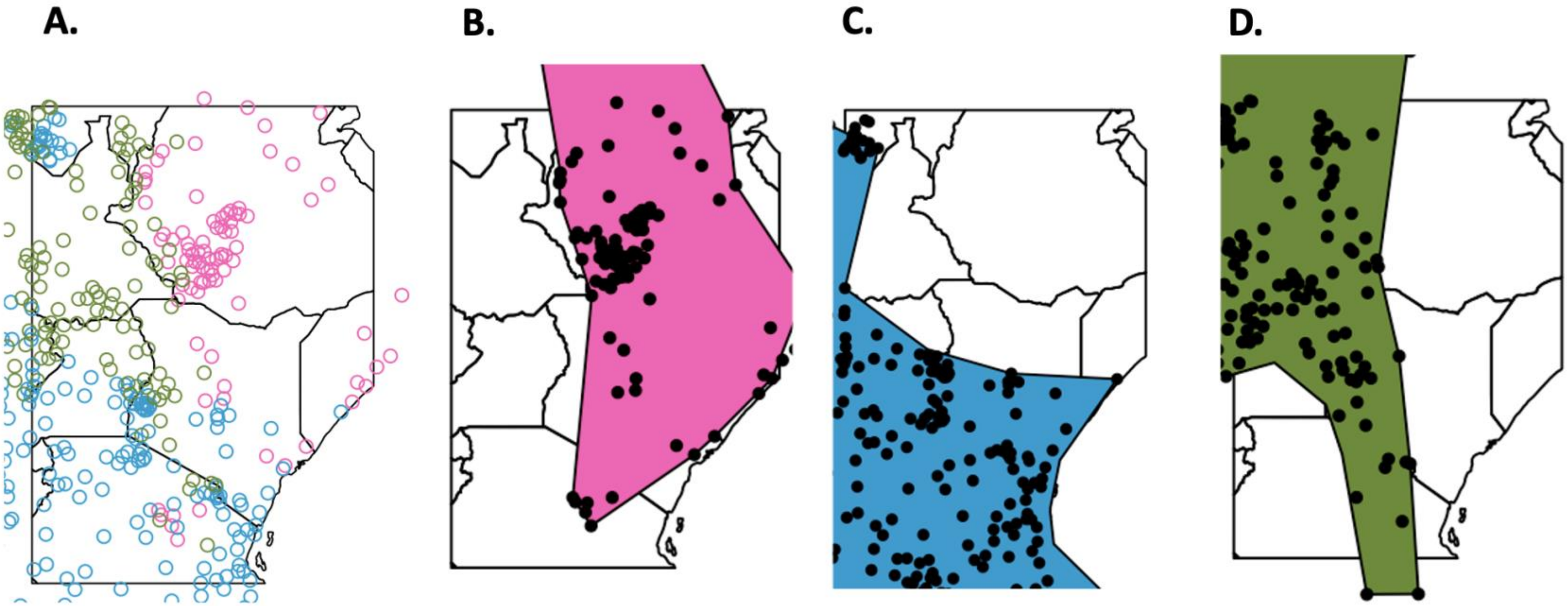

Figure S3: Creation of language family spatial variables. A. Location of the language family coordinates downloaded from Ethiolang and Wikitongues (pink = Afro-Asiatic, blue = Niger-Congo, green = Nilo-Saharan). B-D. Local convex hulls were created for each language family and then merged into one polygon to create categorical presence/absence variables. The final polygons are shown here for each language family, with the coordinates for each language family shown in black.

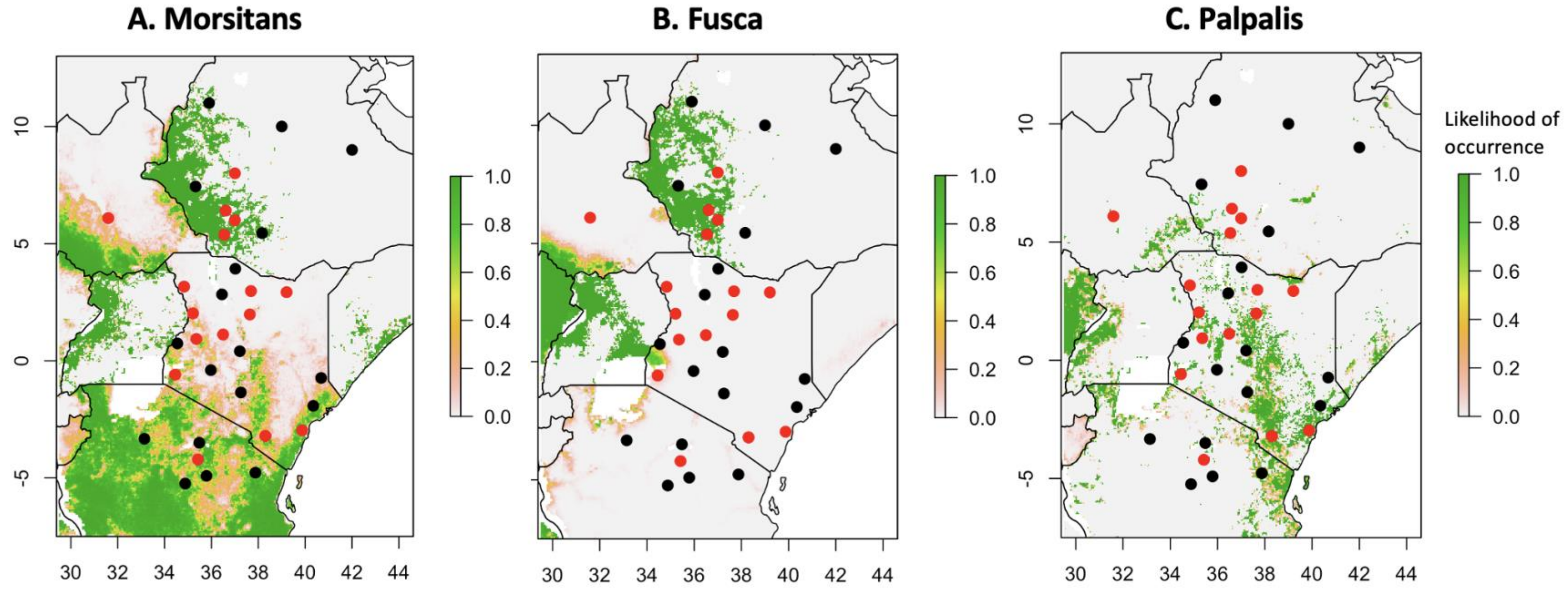

Figure S4: Tsetse suitability maps for the three main group (Morsitans, Fusca, and Palpalis) provided by the Food and Agriculture Organization of the United Nations (Cecchi 2002) and converted to rasters using qGIS 2.18 (QGIS Development Team 2017). Briefly, the morsitans group transmits *T. rhodensiense* in east Africa, the fusca group includes several vectors of livestock trypanosomiasis, and the palpalis group transmits *T. gambiense* in west and central Africa (Bouteille and Dumas 2003). Genetic sampling locations are also shown; points in red practice or recently practiced pastoralism or agropastoralism.

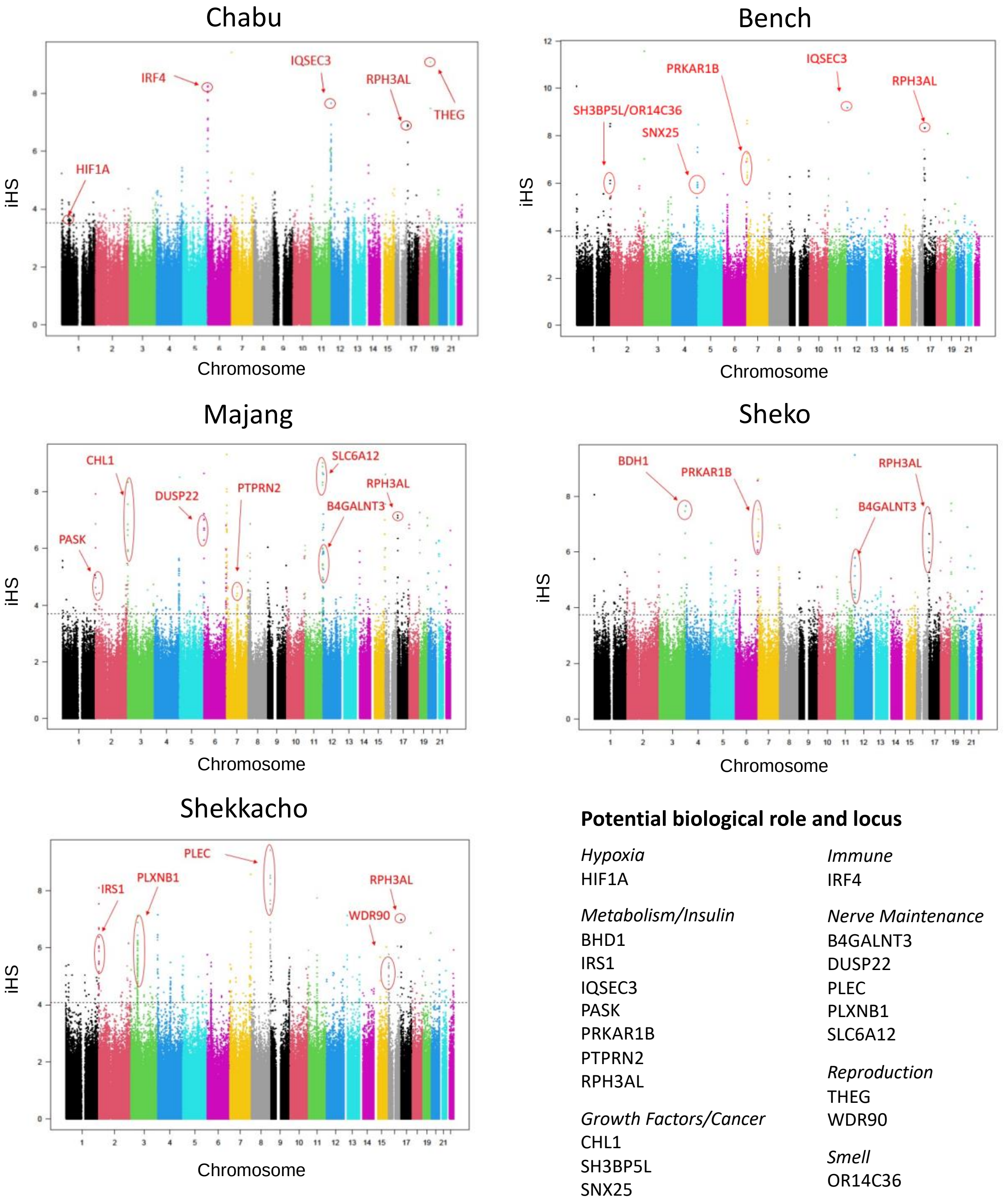

Figure S5: Selection scan results showing absolute value of integrated Haplotype Score (iHS) (Voight et al. 2006) across the genomes for five populations from southwestern Ethiopia. Higher values indicate higher likelihood of positive selection, based on decay of alleles from a query locus. The selection scan was performed with selscan 1.3.0 (Szpiech and Hernandez 2014).

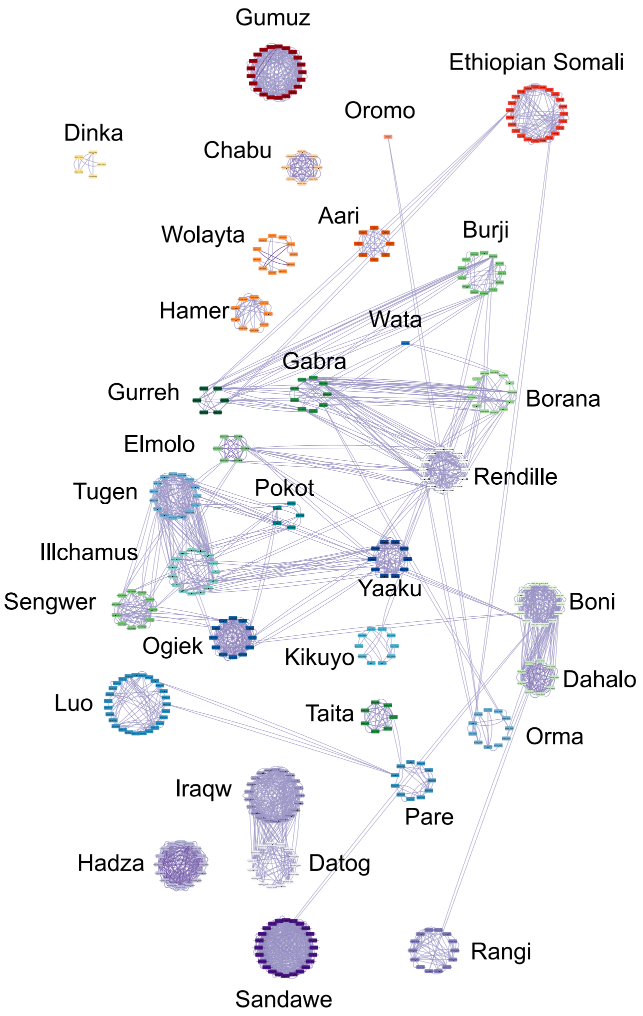

Figure S6: Identical by descent network created in Cytoscape (Shannon et al. 2003). Each individual is a node, and individuals from the same population are arranged in a circle. The locations of the circles correspond to the populations' approximate geographic locations. Edges between the nodes represent the number of shared IBD segments >6cM; however, edges representing <2 segments are not shown. Darker edges shows higher number of shared IBD segments. Individuals who do not share IBD segments with any other individuals are not shown.

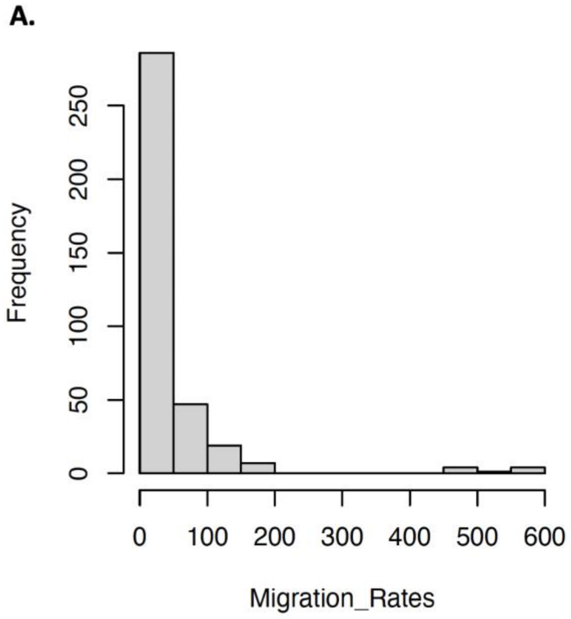

Figure S7: A. Histogram of migration rates from run 1 of MAPS using 2-4cM shared IBD tracts. Migration rates are unbalanced and skewed toward low values. B. By log transforming the migration rates, we obtained a more normal distribution to use as the observed variable in the SPRUCE model.

Dataset S1 (separate file). Candidate genes under positive selection in each of the five southwestern Ethiopia populations. chromosome, position, and for. For each candidate gene, chromosome, position, rsID, and iHS score are also listed (Voight et al. 2006). Candidate genes were determined by taking the top 0.1% largest absolute value iHS scores for each population.

SI References

Al-Asadi H. 2017. plotmaps: Plotting Migration and Population Size Surfaces. R package version 1.0.

Al-Asadi H, Petkova D, Stephens M, Novembre J. 2019. Estimating recent migration and population-size surfaces. *PLoS Genet.* 15:e1007908.

Gopalan S, Berl RE, Myrick JW, Garfield ZH, Reynolds AW, Bafens BK, Belbin G, Mastoras M, Williams C, Daya M. 2022. Hunter-gatherer genomes reveal diverse demographic trajectories during the rise of farming in Eastern Africa. *Curr. Biol.* 32(8): 1852-1860.

Gurdasani D, Carstensen T, Tekola-Ayele F. et al. 2015. The African Genome Variation Project shapes medical genetics in Africa. *Nature* 517:327–332.

Ishwaran H, Kogalur U. 2007. Random survival forests for R. *R News*. 7(2): 25–31.

Ishwaran H, Kogalur U, Blackstone E, Lauer M. 2008. Random survival forests. *Ann. Appl. Statist.* 2(3): 841–860.

Ishwaran H, Kogalur U. 2022. Fast Unified Random Forests for Survival, Regression, and Classification (RF-SRC). R package version 3.0.2.

Pagani L, Kivisild T, Tarekegn A, Ekong R, Plaster C, Romero IG, Ayub Q, Mehdi SQ, Thomas MG, Luiselli D, Bekele E. 2012. Ethiopian genetic diversity reveals linguistic stratification and complex influences on the Ethiopian gene pool. *Am. J. Hum. Genet.* 91(1):83-96.

Shannon P, Markiel A, Ozier O, Baliga NS, Wang JT, Ramage D, Amin N, Schwikowski B, Ideker T. Cytoscape: a software environment for integrated models of biomolecular interaction networks. 2003. *Genome Res*. 13(11):2498-504.

Scheinfeldt LB, Soi S, Lambert C, Ko WY, Coulibaly A, Ranciaro A, Thompson S, Hirbo J, Beggs W, Ibrahim M, Nyambo T. 2019. Genomic evidence for shared common ancestry of East African hunting-gathering populations and insights into local adaptation. *PNAS*. 116(10):4166-4175.

Shannon P, Markiel A, Ozier O, Baliga NS, Wang JT, Ramage D, Amin N, Schwikowski B, Ideker T. Cytoscape: a software environment for integrated models of biomolecular interaction networks. 2003. *Genome Res*. 13(11):2498-504.

Szpiech ZA, Hernandez RD. 2014. selscan: an efficient multithreaded program to perform EHH-based scans for positive selection. *Mol. Biol. Evol.* 31:2824-2827

QGIS Development Team. 2017. QGIS Geographic Information System. Open Source Geospatial Foundation Project.

Voight BF, Kudaravalli S, Wen X, Pritchard JK. 2006. A map of recent positive selection in the human genome. *PLOS Biol*. 4:e72.
